# Worsening of Retinal Detachment after Cataract Surgery in the Eye with Persistent Fetal Vasculature

**DOI:** 10.1155/2021/6641161

**Published:** 2021-02-22

**Authors:** Yuri Aikawa, Takayuki Baba, Tomohiro Nizawa, Hirotaka Yokouchi, Shuichi Yamamoto

**Affiliations:** Department of Ophthalmology and Visual Science, Chiba University Graduate School of Medicine, Chiba, Japan

## Abstract

**Purpose:**

To report a case of persistent fetal vasculature (PFV) with a retinal detachment that worsened after cataract surgery. Pars plana vitrectomy (PPV) was performed which reduced the vitreous traction and reattached the retina. *Observations.* A 20-year-old Myanmarese woman presented with a mature cataract, and her vision was light perception. She underwent uneventful cataract surgery with implantation of an intraocular lens. Her visual acuity improved to 20/200 immediately after the surgery. However, fibrotic tissue was observed between the optic nerve head and the posterior capsule. She was diagnosed with PFV, and she was followed without any intervention. One and a half years after the cataract surgery, she had an advanced retinal detachment which extended over the inferior two quadrants. Her vision deteriorated to 20/400. She underwent PPV, and the PFV tissue was removed which resulted in the reattachment of the retina. The visual acuity improved to 20/60.

**Conclusions:**

Surgeons should be aware that it is possible to worsen a retinal detachment after cataract surgery in the eyes with PFV. A simple technique to release the anterior-posterior traction by the PPV was sufficient to achieve the reattachment of the retina.

## 1. Introduction

Persistent fetal vasculature (PFV) which was previously called persistent hyperplastic primary vitreous (PHPV) is a rare congenital ocular disorder [1]. PFV is characterized by unilateral cataract, persistent vascularized hyaloid, retrolental tissues, microphthalmos, and retinal detachment [2]. PFV is usually diagnosed in childhood because the eyes with PFV usually have poor visual function, leukocoria, and strabismus. These changes alert the parents that the eyes are not normal. Phacoemulsification of the cataract and insertion of an intraocular lens is usually performed on these children, and the postsurgical outcomes are generally good [3].

We have had a case of PFV in an adult Myanmarese woman, and she was initially treated for her advanced cataract with phacoemulsification and implantation of an intraocular lens. After the cataract surgery, there was worsening of the retinal detachment, but we successfully reattached the retina by a simple PPV with removal of the connecting tissue in the retrolental space and optic nerve head.

## 2. Case Report

**Figure 1:**
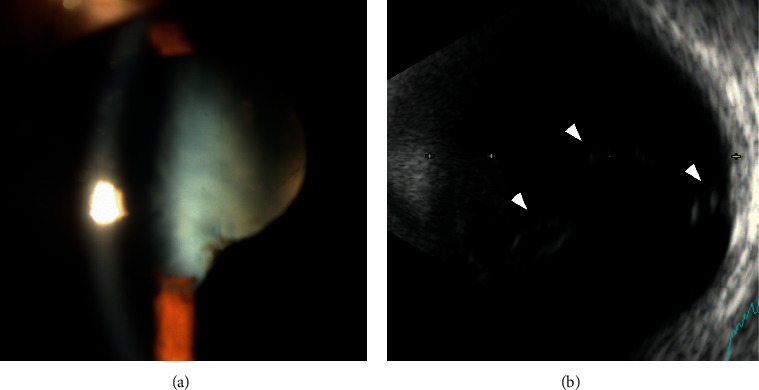
Ocular images of a 20-year-old Myanmarese woman at the initial examination. (a) Slit-lamp image shows a dense cataract in the left eye. Synechia can also be seen at the inferior part of the pupil with no active inflammation. Her visual acuity was light perception in the left eye. (b) B-mode echogram showed fibrous tissue emanating from the optic disc and is attached to the posterior surface of the crystalline lens (arrowheads). No retinal detachment was suspected.

A 20-year-old Myanmarese woman who was aware of a declining vision in her left eye for three years was diagnosed with a mature cataract in her left eye. She was referred to Chiba University Hospital for further examination and treatment for the cataract. She had no medical history of the left eye, although detailed medical records were not available because she had grown up in Myanmar and had recently moved to Japan to receive occupational training. She did not have any systemic disease and abnormalities. The laboratory tests for infectious diseases were negative. She stated that she was born at full-term and had no family history of retinal detachment. Her left vision was light perception at our first examination. She had a mature cataract with a slightly shallow anterior chamber and the absence of inflammation and iris neovascularization. The dense cataract prevented a detailed examination of the fundus, but B-mode echogram showed some strands of high signals extending anteriorly from the optic nerve head (Figure 1). Her right eye was normal with a vision of 20/20. The intraocular pressure was 18 mmHg OD and 18 mmHg OS. The axial length was 23.78 mm in the right eye and 23.46 mm in the left eye.

**Figure 2:**
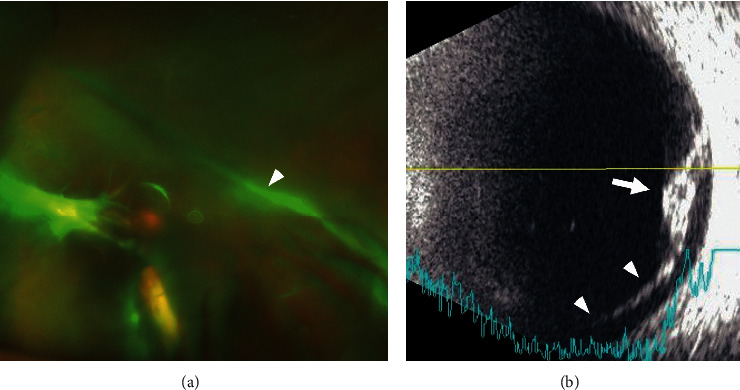
Images of the left eye at one and a half years after the cataract surgery. (a) An ultra-widefield fundus image showing a fibrovascular stalk (arrowhead) which extends from the optic nerve head to the posterior capsule. (b) B-mode echogram showing a fibrovascular tissue (arrow) and a shallow but distinct retinal detachment (arrowheads) at the inferior area.

**Figure 3:**
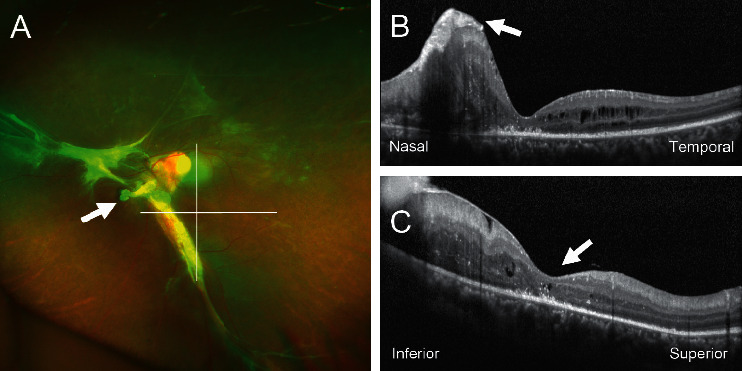
. Images of the left eye at six months after pars plana vitrectomy. (a) An ultra-widefield retinal image showing a stump of the stalk (arrow) that was cut by a vitrectomy cutter. The horizontal and vertical lines through macular indicate the location of tomographic images of B and C. (b) Horizontal optical coherence tomographic (OCT) image showing an attached macula with retinal folds (arrow). Her visual acuity improved to 20/60. (c) Vertical OCT image showing a normal foveal configuration (arrow). The macular is completely attached with some hyperreflective foci, but the outer retinal microstructures are not obvious.

She underwent phacoemulsification and implantation of an intraocular lens in her left eye. The surgery was completed without any complications, and the postoperative course was uneventful with a recovery of the left vision to 20/200. A stalk was observed between the optic nerve and posterior capsule, but the retinal detachment was limited to the retina around the optic nerve. One and a half years after the initial surgery, she noticed a decline of the vision in her left eye, and her visual acuity was 20/400. The area of retinal detachment extended over two quadrants of the inferior retina (Figure 2). There were no retinal breaks. A macula involved detachment was confirmed by optical coherence tomography. PPV with a 27-gauge system was performed, and the stalk was incised with a vitrectomy cutter. Some parts of the stalk were left attached to the optic nerve not to damage the anteriorly stretched retina. No additional procedures such as membrane peeling, drainage of subretinal fluid, and the fluid-air exchange were performed. The posterior vitreous was not separated because the hyaloid was degenerated and attached firmly to the retina. The retinal detachment gradually resolved, and the macula reattachment was confirmed by optical coherence tomography (Figure 3). The vision in her left eye improved to 20/60 and was stable for 19 months after the second surgery without showing any worsening of the retinal proliferation or detachment.

## 3. Discussion

The eyes with PFV are characterized by cataracts, vascularized persistent hyaloid, retrolental tissue, microphthalmos, and retinal detachment. The retinal detachment sometimes presents as a falciform retinal detachment, and it usually extends from the optic nerve to the inferior temporal region. The visual function in the eyes with PFV is usually poor because of the involvement of macula by the retinal detachment, severe macular deformity, and refractory glaucoma. Despite recent extensive surgical interventions and visual rehabilitations, the treatment of PFV is still challenging. Dass et al. reported that the final visual acuity was better than 20/800 6 in 35 eyes (17%) [4], and Alexandrakis et al. reported that 14 in 30 eyes (47%) achieved a final visual acuity of 20/400 or better [5].

We presented a PFV case with a visual decrease after cataract surgery and visual improvement after PPV. Before the cataract surgery, we could not diagnose the PFV because of the poor visibility of fundus caused by a fibrovascular tissue that extended from optic nerve head to posterior lens capsule. We speculated that this PFV tissue caused the anterior-posterior traction on the optic disc and surrounding retina to cause the development of the retinal detachment. Previous histological studies reported that the PFV stalk contained vascular tissues and fibrovascular connective tissue with mesodermal hyperplasia with smooth muscle actin [6]. These tissues exerted contractile force when they regressed. Our second approach by PPV was effective in reducing retinal detachment because we released the traction by cutting the stalk. Another option was posterior capsulotomy and anterior vitrectomy to remove retrolental tissue [3]. However, we were not confident of the intraoperative diagnosis of PFV and avoided further invasive procedures.

The characteristics of the OCT findings of PFV have been well summarized [7]. The retinal layer structure near the falciform retinal detachment was relatively well maintained except for the peripheral temporal region which showed marked thinning and disorganized retinal layer structures. In our case, the foveal configuration was well preserved after the absorption of subretinal fluid, and it was associated with relatively good postoperative vision. The restoration of external limiting membrane and ellipsoid zone could not be confirmed probably because of the chronic retinal detachment.

In conclusion, we experienced a PFV case that had worsening of retinal detachment after uneventful cataract surgery. The retinal detachment was successfully treated by simple vitrectomy. We suggest that the vasoproliferative tissue played a role in the development and resolution of retinal detachment. Although PPV was effective, posterior capsulotomy with anterior vitrectomy is one option to prevent worsening of the retinal detachment.

## Data Availability

No data were used to support this study.
